# Uneven attention to climate tipping points

**DOI:** 10.1073/pnas.2618666123

**Published:** 2026-09-28

**Authors:** Heming Wang, Bingyu Chen, Fengmei Ma, Nico Wunderling, Timothy M. Lenton, Asaf Tzachor

**Affiliations:** ^a^ Key Laboratory of Eco-Industry (Ministry of Ecology and Environment), School of Metallurgy, Northeastern University, Shenyang, Liaoning 110819, China; ^b^ Commonwealth Scientific and Industrial Research Organisation, Canberra, ACT 2601, Australia; ^c^ Center for Collective Learning, Corvinus Institute for Advanced Studies, Corvinus University of Budapest, Budapest 1093, Hungary; ^d^ State Key Laboratory of Regional and Urban Ecology, Institute of Urban Environment, Chinese Academy of Sciences, Xiamen 361021, China; ^e^ Potsdam Institute for Climate Impact Research, Member of the Leibniz Association, Potsdam 14473, Germany; ^f^ Center for Critical Computational Studies, Goethe University Frankfurt, Frankfurt am Main 60629, Germany; ^g^ Senckenberg Research Institute and Natural History Museum, Member of the Leibniz Association, Frankfurt am Main 60325, Germany; ^h^ Global Systems Institute, University of Exeter, Exeter EX4 4QE, United Kingdom; ^i^ School of Sustainability, Aviram Sustainability and Climate Program, Reichman University, Herzliya 4610101, Israel; ^j^ Institute for Technology and Humanity, Centre for the Study of Existential Risk, University of Cambridge, Cambridge CB2 1SB, United Kingdom

**Keywords:** sustainability science, climate tipping points, climate policy

## Abstract

Climate tipping points have moved from theoretical concern to a practical challenge for climate-risk assessment and governance. Yet whether scientific attention is aligned with risk-relevant characteristics of climate tipping elements (CTEs) has not been systematically assessed. Across 20,736 CTE-specific records spanning 2000–2025 and addressing 14 tipping elements, attention was concentrated on the Greenland Ice Sheet and Atlantic Meridional Overturning Circulation, while Boreal Permafrost (abrupt thaw) and the North Atlantic Subpolar Gyre received comparatively limited attention despite their relatively low estimated warming thresholds. Explicit engagement with risk-relevant tipping dynamics, including abrupt transitions, hysteresis, or irreversibility, was also limited. These patterns may influence which tipping risks can be most readily assessed, prioritized, and governed.

Climate tipping points (CTPs) in the Earth system represent critical thresholds beyond which change in a climate tipping element (CTE) can become self-perpetuating (1, 2). These nonlinear dynamics pose cascading risks to global climate stability and human societies (3–5) and have transitioned from theoretical constructs to central concerns in climate policy debates (6, 7).

Even as scientific understanding of CTPs has advanced, it remains unclear whether scientific attention is distributed in relation to their risk-relevant characteristics. We hypothesized that lower estimated warming thresholds would not systematically correspond to greater scientific attention across CTEs and that explicit engagement with risk-relevant tipping dynamics would remain limited.

To evaluate these hypotheses, we analyze 130,922 publications from 2000–2025, and identify 20,736 publication records directly addressing one of 14 prespecified CTEs drawn from the assessment of Armstrong McKay et al. (2). Following its Earth-system-domain classification, these comprised nine cryosphere elements—boreal permafrost abrupt thaw, boreal permafrost collapse, mountain glaciers, the Greenland, West Antarctic, and East Antarctic ice sheets, East Antarctic subglacial basins, Arctic winter sea ice, and Barents Sea winter ice, two ocean-atmosphere circulation elements—the Atlantic Meridional Overturning Circulation and Labrador-Irminger Seas convection (North Atlantic Subpolar Gyre), and three biosphere elements—low-latitude coral reefs, the Amazon rainforest, and Sahel vegetation and the West African monsoon. This prespecified set spans multiple Earth-system domains but does not encompass all proposed ecosystem tipping points or the broader literature on ecological regime shifts (8). We quantify the distribution and temporal development of scientific attention across these CTEs and compare it with Earth-system domain, estimated global-warming thresholds (e.g., tipping at 2 °C vs. 4 °C), and explicit engagement risk-relevant tipping dynamics, including abrupt transitions, hysteresis, and irreversibility. By treating scientific attention as an empirical property of the knowledge system, we test whether research effort is aligned with risk-relevant characteristics of tipping elements and with central dimensions of tipping behavior.

## Results

Scientific attention is unevenly distributed across all dimensions examined, yielding a highly concentrated pattern of knowledge accumulation. These asymmetries may shape which tipping risks are most readily characterized, compared, and incorporated into anticipatory assessment and policy (9, 10).

Our analysis reveals disparities in scholarly attention across Earth system domains. While cryosphere-related elements comprise 9 of the 14 core and regional CTEs identified in literature (2), they account for less than half (44.8%) of total scientific publications from 2000 to 2025. Furthermore, within the cryosphere domain, research is unevenly distributed. The Greenland Ice Sheet has attracted substantial attention, accounting for 4,141 publications (20.0% of the total). In contrast, the other eight cryosphere CTPs have received comparatively limited scientific attention, each representing less than 7% of total publications. The most pronounced gaps are observed for the East Antarctic Subglacial Basins (just 56 publications over 26 y; 0.3%), Boreal Permafrost (abrupt thaw) (139; 0.7%), and Barents Sea Winter Ice (224; 1.1%).

**Fig. 1.**
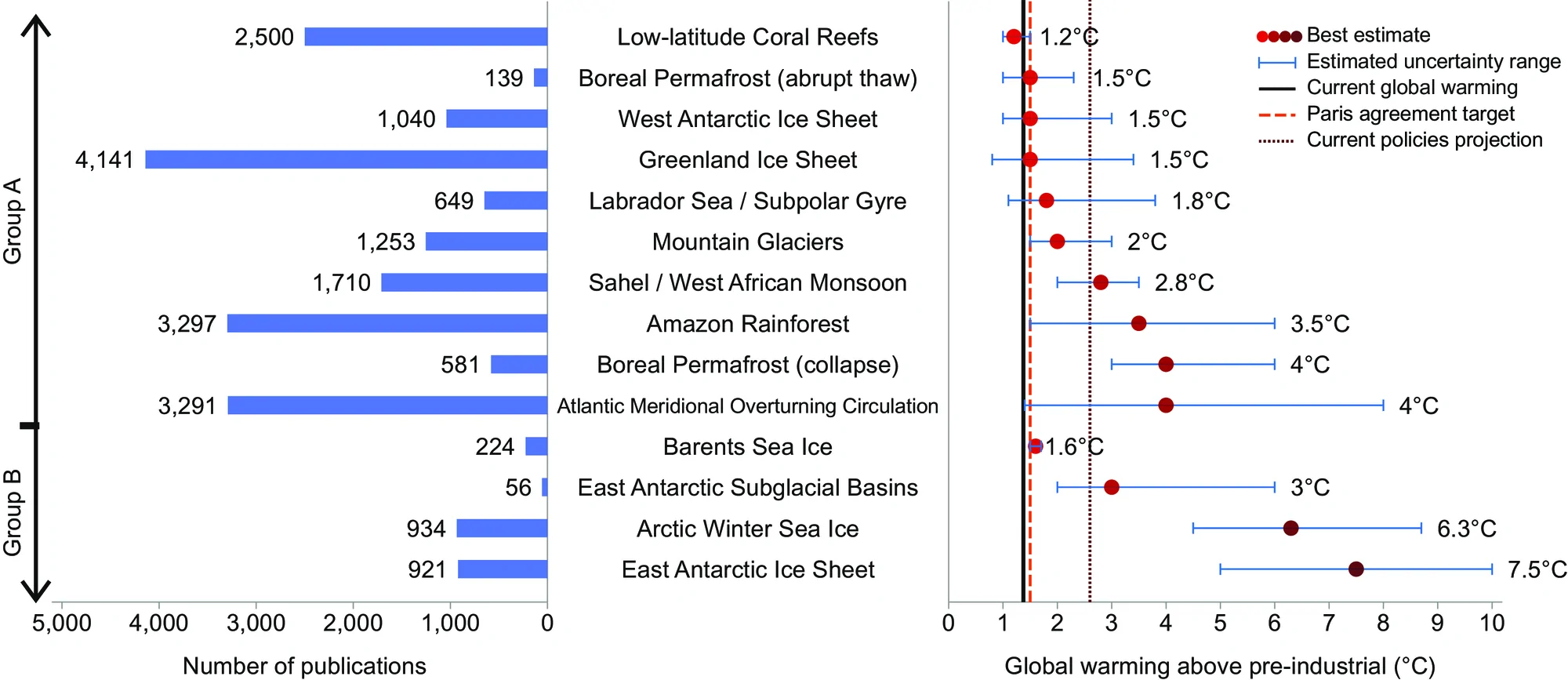
Scientific attention and estimated global-warming thresholds across 14 CTEs, 2000–2025. Bars in the *Left* panel indicate the total number of publications associated with each CTE. In the *Right* panel, horizontal blue lines show the estimated uncertainty range of each CTE’s global-warming-level threshold, and overlaid dots indicate the corresponding best estimates. The black shaded band marks the estimated 2024 global warming level of 1.34 to 1.41 °C above preindustrial levels (11), the orange dashed line marks the Paris Agreement limit of 1.5 °C (12), and the red dotted line marks projected warming of 2.6 °C by 2100 under current policies (13). Group A comprises CTEs assessed in the Global Tipping Points Report 2025 risk register; Group B comprises CTEs not assessed in that risk register. Threshold estimates and warming levels are expressed relative to the preindustrial baseline (2, 7).

These disparities are concerning when viewed in relation to warming thresholds (Fig. 1). As of 2024, global mean temperature has reached an estimated 1.34 to 1.41 °C above preindustrial levels. This places at least six CTPs at or beyond minimum estimates of their temperature thresholds. Despite these threshold ranges overlapping current warming, several of these tipping elements remain underrepresented in the literature, including Boreal Permafrost (abrupt thaw) and the North Atlantic Subpolar Gyre which received only 0.7% and 3.1% of all CTP related publications, respectively.

**Fig. 2.**
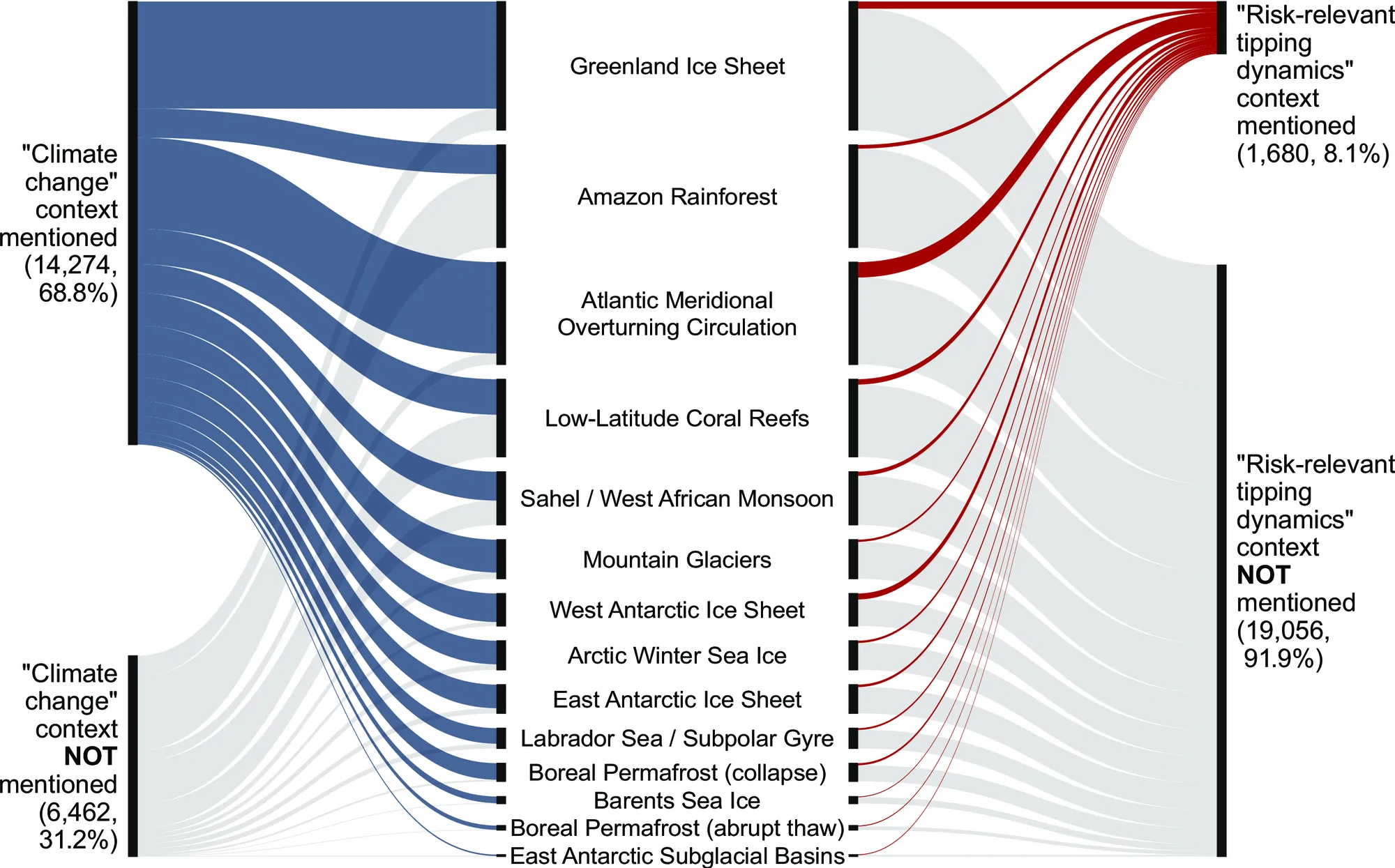
Climate-change framing and engagement with risk-relevant tipping dynamics across 14 CTEs. For each CTE, the figure shows the number of publications classified as explicitly addressing climate change (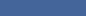) and the number classified as engaging with risk-relevant tipping dynamics (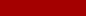). Gray segments (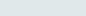) indicate publications not assigned to the corresponding category. Classification criteria, treatment of uncertain cases, and validation procedures are described in the *SI Appendix*.

Despite mounting interest in global warming and tipping dynamics, substantial asymmetries persist in how tipping elements are framed in relation to climate change (Fig. 2). Several CTEs with relatively low estimated global-warming-level thresholds have received comparatively limited scientific attention. Boreal Permafrost (abrupt thaw) appears in only 132 publications (0.9% of the climate-framed corpus) over 26 y, the West Antarctic Ice Sheet features in 914 publications (6.4%), and low-latitude coral reefs, despite being among the first confirmed tipping elements to cross their thermal threshold (7), appear in 1,139 publications (8.0%) over the same period.

Risk-relevant tipping dynamics encompass processes affecting the abruptness, self-perpetuation, persistence, or reversibility of tipping transitions. Yet explicit engagement with these dynamics remains limited in the scientific literature (Fig. 2). Across the entire corpus, only 8.1% of publications engage directly with risk-relevant tipping dynamics. Within this subset, most CTEs are represented by comparatively small publication literatures. Boreal Permafrost (abrupt thaw), for instance, appears in just 22 publications over 26 y, despite the fact that irreversible thaw could significantly amplify global warming through greenhouse gas feedbacks (14). Low-latitude coral reefs appear in 168 publications over the same period. For the other CTEs whose threshold estimates fall within current warming levels, attention to risk-relevant tipping dynamics varies considerably, from 491 publications on the Atlantic Meridional Overturning Circulation (AMOC) to a mere 53 on the North Atlantic Subpolar Gyre.

## Discussion

Unequal publication volumes across CTEs are neither unexpected nor inherently undesirable. The relevant analytical questions are therefore whether scientific attention aligns with estimated global-warming-level thresholds and whether risk-relevant tipping dynamics are explicitly examined. Accordingly, our analysis characterizes the distribution of research activity; it does not assess the validity of individual tipping-point claims or the maturity of evidence for each CTE. The observed patterns may reflect differences in observability, data availability, disciplinary traditions, and institutional path dependence—mechanisms that warrant dedicated investigation beyond the present study.

Against this background, we find no systematic tendency for CTEs with lower estimated global-warming-level thresholds to receive greater scientific attention. Boreal Permafrost (abrupt thaw), the North Atlantic Subpolar Gyre, and the West Antarctic Ice Sheet receive comparatively limited attention despite their relatively low estimated thresholds, whereas the Greenland Ice Sheet, with a similarly low estimated threshold, accounts for the largest share of publications. The comparatively small literature identified for the North Atlantic Subpolar Gyre should be interpreted cautiously, because its dynamics are closely coupled to—and are sometimes examined within—the broader literature on the Atlantic Meridional Overturning Circulation rather than treated as a distinct tipping element. Its publication count may therefore not capture all relevant research.

Explicit engagement with irreversibility, hysteresis, self-perpetuating dynamics, and other risk-relevant aspects of tipping behavior remains limited across much of the CTP literature. For instance, the Boreal Permafrost (abrupt thaw) and low-latitude coral reefs, both of which are already experiencing threshold-exceeding impacts, are seldom studied through the lens of collapse or dieback. This reflects a reluctance to engage with high-consequence, uncertain futures.

Overshooting 1.5 °C of global warming is now widely regarded as inevitable (15), increasing the likelihood of crossing multiple CTPs (2). If current trajectories continue, global temperatures are projected to rise by 2.6 °C by the end of the century (13). At that level, the question is no longer whether key CTPs will be crossed, but when. In this context, science must anticipate risk-relevant tipping dynamics while also closing critical knowledge gaps that shape perceptions of tipping likelihood, as well as strategies for prevention and response. Addressing asymmetries—across tipping elements and research framings—is essential for informing policy, from the global to local scales.

Our analysis further suggests that some CTEs face a two-tiered knowledge gap: They remain unassessed in current policy-facing risk registers and comparatively sparsely studied in the scientific literature, limiting both anticipatory governance and evidence-based prioritization.

These findings are not a prescription for individual researchers to abandon established areas of expertise. Rather, they may inform portfolio-level decisions by funding agencies, research institutions, and collaborative programs when developing new initiatives or expanding observational and modeling capacity. Targeted support for observation, modeling, and synthesis could strengthen the knowledge base for sparsely studied CTEs with relatively low estimated global-warming-level thresholds, including Boreal Permafrost (abrupt thaw), the West Antarctic Ice Sheet, and the North Atlantic Subpolar Gyre. Greater attention to the element-specific dynamics that govern abruptness, self-perpetuation, persistence, and irreversibility should likewise be fostered across both established and emerging research programs.

## Materials and Methods

We focused on 14 CTEs to test whether scientific attention aligned with estimated warming thresholds and other risk-relevant characteristics. We compiled English-language journal articles and reviews published between 2000 and 2025 and indexed in Web of Science, Scopus, or OpenAlex; other nonscholarly publication types were excluded. We used a staged large language model pipeline to screen abstracts and classify framing contexts consistently at corpus scale. For the risk-relevant-tipping-dynamics classification, we assessed four overlapping semantic families: I) abrupt system responses; II) self-perpetuating, self-reinforcing, or self-sustaining dynamics and feedback; III) persistent alternative states or regimes, including alternative stable states and bistability; and IV) hysteresis or reversibility constraints, including irreversibility. Three prompt families were implemented with DeepSeek-V3.2-Exp and DeepSeek-V4-Pro, under deterministic settings and validated against held-out reference sets independently annotated by two coauthors. Uncertain classifications were manually reviewed and resolved before aggregation. We assessed sensitivity to literature coverage by repeating the attention analysis separately across the three source databases and within a selective journal subset. Publication counts were interpreted as measures of scientific attention, not as direct indicators of evidentiary quality or knowledge maturity. The details are provided in *SI Appendix*.

## Supplementary Material

Appendix 01 (PDF)

Dataset S01 (XLSX)

## Data Availability

Search queries, raw data, prompts, and results have been deposited in Zenodo (https://zenodo.org/records/21476189) (16).
