# Supplementary material for: Uneven attention to climate tipping points
Source: Proc Natl Acad Sci U S A. 2026 Sep 28;123(40):e2618666123. doi: 10.1073/pnas.2618666123 (PMC13643155; doi:10.1073/pnas.2618666123)
Supplement: Supplementary file 1 [file pnas.2618666123.sapp.pdf]

Supporting Information for
Uneven attention to climate tipping points

Heming Wang, Bingyu Chen, Fengmei Ma, Nico Wunderling, Timothy M. Lenton and Asaf Tzachor

Corresponding Authors: Heming Wang; Fengmei Ma; Asaf Tzachor
Email: wanghm@mail.neu.edu.cn; fmma@iue.ac.cn; atzachor@runi.ac.il

This PDF file includes:

Supporting text

Data, Materials, and Software Availability. The Supplementary Dataset, which details the analytical workflows and evaluation outputs, has been deposited in the Zenodo repository at https://zenodo.org/records/21476189.

Extended Methods

Data sources and retrieval

Bibliographic records were retrieved from the Web of Science (WoS), Scopus, and OpenAlex databases. The initial search was conducted on 20 August 2025 and covered literature published between 1 January 2000 and 31 December 2024. This search was subsequently updated on 5 February 2026 to incorporate records published during the full year of 2025. No geographic restrictions were applied, ensuring a global scope for the literature search.

Data preprocessing

A total of 33,793 records were retrieved from WoS, 39,136 from Scopus, and 57,993 from OpenAlex. To construct a high-quality analytical corpus, all retrieved records underwent the following multi-step preprocessing and filtering pipeline:

- Step 1: Publication filtering (17,384 records removed). We retained only English-language records classified as articles or reviews and published between 2000 and 2025.
- Step 2: Removal of invalid records (19,989 records removed). We excluded retracted publications, records with missing titles or abstracts, and abstracts containing placeholder text (e.g., "an abstract is not available for this content").
- Step 3: Text normalization (74 records removed). DOI, title, and abstract fields were normalized by converting text to lowercase, removing excess whitespace and special characters, deleting copyright or disclaimer statements, and stripping the leading "Abstract" label from the abstract field. Abstracts shorter than 20 words were excluded.
- Step 4: Deduplication (49,506 records removed). Records were first deduplicated using exact DOI matches. For records lacking a DOI, duplicate candidates were identified using character-level trigram similarity of normalized titles, with records treated as duplicates when title similarity was at least 0.9 and publication years differed by no more than one year.

Following preprocessing and filtering, the final analytical corpus comprised 43,969 publications.

Training and validation set construction

To support prompt refinement and independent performance evaluation, we constructed training and validation sets before applying the screening pipeline to the full corpus. For each prompt-based task in the main screening pipeline, samples were allocated to training and validation sets using a target training-to-validation ratio of approximately 2:1. For CTE identification, climate-change context classification, and risk-relevant tipping dynamics context classification, each training set contained approximately 280–330 manually reviewed samples, and each validation set contained approximately 140–180 samples. Training sets were used for iterative prompt refinement, whereas validation sets were reserved for final performance evaluation. To ensure that the selected samples represented the semantic range of the corpus,

the full corpus was first ordered by semantic similarity to predefined questions using the Qwen-text-embedding-v4 model; samples were then selected at equal intervals from the ordered list.

Manual validation and performance evaluation. To establish reliable reference labels for iterative prompt revision and independent performance evaluation, we implemented a rigorous manual blind-review process across all training and validation sets. Following an initial judgment by one researcher, two independent reviewers annotated the same samples in parallel without access to one another's labels. Discrepancies were resolved by consensus. The reviewed training labels were used to support iterative prompt revision, whereas the reviewed validation labels were reserved for final performance evaluation.

Prompt design

To identify literature referring to the 14 CTEs and to classify the framing contexts referenced in publication abstracts, we designed three prompt families: one for CTE identification and two for framing-context classification. We then applied them in a staged screening pipeline. Abstract analysis was performed for CTE identification and climate-change context classification using the DeepSeek-V3.2-Exp large language model, selected for its long-context processing capacity (up to a 128k-token context window). The DeepSeek-V4-Pro model (up to a 1M-token context window) was used for identifying risk-relevant tipping dynamics. To improve reproducibility and reduce stochastic variation during the screening process, all model requests were executed with a temperature setting of 0.0, with all other API hyper-parameters (e.g., top_p) strictly retained at their default values.

The identification and screening pipeline consisted of two steps:

- Step 1: CTE identification. We developed a first set of prompts to determine whether an abstract mentions any of the 14 CTEs. Prompts followed a standardized template and XML structure; the <Terms> field was customized for each CTE.
- Step 2: Context classification. For abstracts classified in Step 1 as mentioning a given CTE, two additional prompt sets were applied to identify whether the abstract discussed the CTE in the context of (i) climate change or (ii) risk-relevant tipping dynamics. These prompts used the same overall template structure, while the <Steps> and <Notes> fields were tailored for CTEs requiring more complex adjudication.

Following Steps 1–2, 20,666 records were retained for downstream analysis.

Boreal Permafrost handling. For Boreal Permafrost, publications were further classified by degradation mode – regional (abrupt thaw) versus global (systemic collapse) – to reflect differences in associated tipping thresholds. To construct representative training and validation sets for this additional classification, we applied the same semantic-similarity sorting and equal-interval sampling strategy described above, so that the selected records spanned the full semantic range of Boreal Permafrost abstracts. This additional degradation-mode classification used a smaller manually reviewed set, comprising approximately 60 training samples and 30 validation samples. Publications addressing both modes (n = 70) were counted in both categories. From a total of 650 Boreal Permafrost publications, this yields 581 assigned to the global mode (511 exclusive + 70 overlapping) and 139 assigned to the regional mode (69 exclusive + 70 overlapping). Accordingly, downstream screening and analysis were conducted

on 20,736 records (20,666 + 70 overlapping assignments).

Record counts across CTEs. Literature retrieval, screening, and subsequent analyses were conducted separately for each of the 14 CTEs using CTE-specific search queries. Publications addressing more than one CTE were included in each relevant CTE-specific dataset. Accordingly, the 20,666 records retained after CTE identification and context classification, and the 20,736 records included after Boreal Permafrost degradation-mode assignment, represent the summed records across the CTE-specific datasets rather than counts of unique publications.

Evaluation of prompt-based screening performance

Prompt-based screening performance was evaluated by comparing the LLM screening outputs with the manually reviewed reference labels in the training and validation sets. For the prompt-based classifications used in the main screening pipeline (i.e., CTE identification, climate-change context classification, and risk-relevant tipping dynamics context classification), performance was evaluated separately using accuracy, precision, recall, and F1 score:

$$Accuracy = \frac{TP + TN}{TP + FP + TN + FN} \tag{1}$$

$$Precision = \frac{TP}{TP + FP} \tag{2}$$

$$Recall = \frac{TP}{TP + FN} \tag{3}$$

$$F_1 = 2 \times \frac{Precision \times Recall}{Precision + Recall} \tag{4}$$

Here, *TP* and *TN* denote the numbers of correctly classified positive and negative samples, respectively. *FP* denotes the number of negative samples incorrectly classified as positive, and *FN* denotes the number of positive samples incorrectly classified as negative. On the held-out validation set for the CTE relevance screening task, accuracy, precision, recall, and F1 score were 0.979, 0.985, 0.971, and 0.978, respectively. For the climate change context classification task, the corresponding values were 0.956, 0.929, 0.987, and 0.957, respectively. For the classification of explicit engagement with risk-relevant tipping dynamics, they were 0.895, 0.807, 0.971, and 0.882, respectively.

For the Boreal Permafrost degradation-mode classification, performance evaluation required a separate procedure because each sample was assigned to one of three reference categories: global-only degradation, regional-only degradation, or both modes. We converted this three-category task into three binary comparisons. In each one-versus-rest comparison, samples assigned to global-only degradation, regional-only degradation, or both modes were treated in turn as the positive class, while samples assigned to the remaining two categories were combined as the negative class. For each category *k*, category-specific precision, recall, and F1 score were first calculated as:

$$Precision_k = \frac{TP_k}{TP_k + FP_k} \tag{5}$$

$$Recall_k = \frac{TP_k}{TP_k + FN_k} \quad (6)$$

$$F_{1,k} = 2 \times \frac{Precision_k \times Recall_k}{Precision_k + Recall_k} \quad (7)$$

Here, k denotes one of the three Boreal Permafrost reference categories, and TP_k, FP_k, and FN_k denote the corresponding true-positive, false-positive, and false-negative counts from the category-specific one-versus-rest comparison.

Precision, recall, and F1 score were then reported as macro-averaged metrics across the three category-specific comparisons:

$$Precision_{macro} = \frac{1}{K}\sum_{k=1}^{K} Precision_k \quad (8)$$

$$Recall_{macro} = \frac{1}{K}\sum_{k=1}^{K} Recall_k \quad (9)$$

$$F_{1,macro} = \frac{1}{K}\sum_{k=1}^{K} F_{1,k} \quad (10)$$

Here, $K = 3$, corresponding to global-only degradation, regional-only degradation, and both modes.

Accuracy for the Boreal Permafrost degradation-mode classification was calculated as the overall proportion of correctly classified samples and was not macro-averaged:

$$Accuracy_{BP} = \frac{N_{correct}}{N_{total}} \quad (11)$$

Here, $N_{correct}$ is the number of samples whose predicted category matched the reference category, and N_{total} is the total number of labeled samples for this task. Accuracy for the Boreal Permafrost degradation-mode classification was calculated directly from the three reference categories – global-only degradation, regional-only degradation, and both modes – as the proportion of samples whose predicted category matched the reference category. This one-versus-rest evaluation procedure was used to assess degradation-mode classification performance; in the downstream analysis, publications classified as addressing both modes were counted in both the global and regional degradation categories. For this degradation-mode classification, validation accuracy, macro-averaged precision, macro-averaged recall, and macro-averaged F1 score were all above 0.91.

Sensitivity to database coverage and journal scope

The literature on climate tipping elements spans heterogeneous conceptual definitions, methodological approaches, and forms of evidence. Individual publications should therefore not be treated as equivalent units of evidentiary strength, and publication counts should not be interpreted as direct measures of

evidence quality or knowledge maturity. Our analysis instead treats eligible scientific publications as observable records of research activity and examines the relative distribution of scholarly attention across CTEs. We therefore tested whether the observed distribution was sensitive to bibliographic database coverage or journal scope.

We repeated the attention analysis separately for records retrieved from Web of Science, Scopus, and OpenAlex, as well as for the merged deduplicated corpus. We additionally constructed a selective journal subset comprising 28 Q1 journals in the JCR 2025 *Meteorology & Atmospheric Sciences* category and five multidisciplinary journals: *Nature*, *Science*, *Proceedings of the National Academy of Sciences*, *Nature Communications*, and *Science Advances*. This subset was used to assess sensitivity to journal scope and was not interpreted as an article-level appraisal of evidentiary quality.

Within each corpus, the publication share associated with CTE i was calculated as:

$$p_i = \frac{n_i}{\sum_{j=1}^{14} n_j} \tag{12}$$

where P_i denotes the publication share associated with CTE i, and n_i denotes the number of publications assigned to that CTE. Because publications could be assigned to more than one CTE, the denominator represents the total number of CTE–publication associations rather than unique publications.

The three database-specific distributions were strongly concordant with the merged corpus (Spearman's ρ = 0.982–0.996). The Greenland Ice Sheet, Amazon Rainforest, and Atlantic Meridional Overturning Circulation ranked among the three most frequently studied CTEs in all four corpora. The selective journal subset also retained the broader concentration of attention on these elements (Spearman’s ρ= 0.846), although low-latitude coral reefs showed a marked scope-dependent difference. This exception likely reflects the disciplinary composition of the journal subset and indicates that individual CTE estimates may remain sensitive to journal scope.

These comparisons show that the principal concentration pattern was not attributable to any single bibliographic database and remained evident under a narrower journal scope. They do not establish the evidentiary quality of individual publications or imply that all element-specific publication shares are invariant to corpus composition. The analysis therefore supports use of the merged corpus to characterize broad patterns in the allocation of scientific attention while preserving appropriate caution regarding article-level quality and scope-sensitive differences among individual CTEs.
